# Understanding the Potential and Risk of Bacterial Siderophores in Cancer

**DOI:** 10.3389/fonc.2022.867271

**Published:** 2022-06-17

**Authors:** Valentina Pita-Grisanti, Kaylin Chasser, Trevor Sobol, Zobeida Cruz-Monserrate

**Affiliations:** ^1^ The Ohio State University Interdisciplinary Nutrition Program, The Ohio State University, Columbus, OH, United States; ^2^ Division of Gastroenterology, Hepatology, and Nutrition, Division of Internal Medicine, The Ohio State University Wexner Medical Center, Columbus, OH, United States; ^3^ The Comprehensive Cancer Center–Arthur G. James Cancer Hospital and Richard J. Solove Research Institute, The Ohio State University, Columbus, OH, United States

**Keywords:** microbiome, bacteria, siderophores, enterobactin, deferoxamine, cancer, tumor, iron

## Abstract

Siderophores are iron chelating molecules produced by nearly all organisms, most notably by bacteria, to efficiently sequester the limited iron that is available in the environment. Siderophores are an essential component of mammalian iron homeostasis and the ongoing interspecies competition for iron. Bacteria produce a broad repertoire of siderophores with a canonical role in iron chelation and the capacity to perform versatile functions such as interacting with other microbes and the host immune system. Siderophores are a vast area of untapped potential in the field of cancer research because cancer cells demand increased iron concentrations to sustain rapid proliferation. Studies investigating siderophores as therapeutics in cancer generally focused on the role of a few siderophores as iron chelators; however, these studies are limited and some show conflicting results. Moreover, siderophores are biologically conserved, structurally diverse molecules that perform additional functions related to iron chelation. Siderophores also have a role in inflammation due to their iron acquisition and chelation properties. These diverse functions may contribute to both risks and benefits as therapeutic agents in cancer. The potential of siderophore-mediated iron and bacterial modulation to be used in the treatment of cancer warrants further investigation. This review discusses the wide range of bacterial siderophore functions and their utilization in cancer treatment to further expand their functional relevance in cancer detection and treatment.

## 1 Introduction

**Figure 1:**
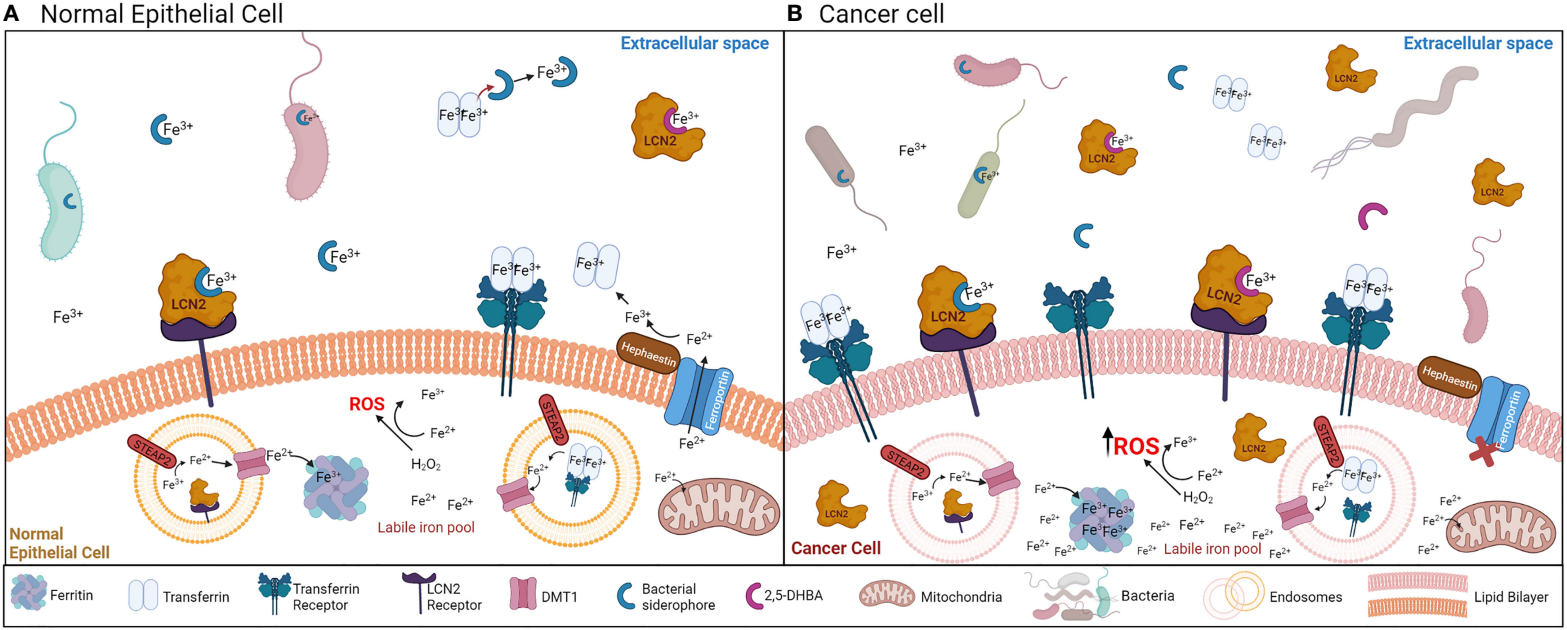
**(A)** Mechanisms of iron acquisition between bacteria and normal epithelial cells. Bacteria-secreted siderophores and mammalian siderophores (2,5-DHBA) acquire ferric iron (Fe^3+^) for bacteria or host uptake. Siderophores can also chelate iron away from transferrin. LCN2 can bind the bacteria-secreted siderophores to sequester ferric iron (Fe^3+^) away from bacteria. Transferrin and LCN2 bind ferric iron in the extracellular space, and by binding the transferrin receptor or the LCN2 receptor (respectively) in the cell surface, the transferrin/LCN2-iron complex enters the cell through endocytosis. Once iron is in the cytoplasm, it is converted to the ferrous form (Fe^2+^) by the STEAP2 enzyme, and exits the endosome through the DMT1 transporter. In the cytosol, iron can be stored in ferritin back in the ferric form (Fe^3+^). Iron can exit the cell *via* ferroportin, which is regulated by hephaestin. This process is tightly regulated to avoid the generation of ROS from the labile iron pool (free ferrous iron (Fe^2+^) in the cytoplasm). **(B)** Mechanisms of iron acquisition between bacteria and cancer cells. Many cancers are characterized by increased bacterial growth and dysbiosis. Iron uptake is increased in cancer cells, which is accomplished by increasing the function of transferrin, the transferrin receptor, ferritin iron storage, and decreasing the function of ferroportin. LCN2 and its receptor are also increased during cancer. Increased ferrous iron (Fe^2+^) accumulation in the cytosol generates ROS.

Siderophores are iron chelating molecules produced by nearly all organisms to enhance iron acquisition from the environment (1–3). Iron is an essential micronutrient for biological and metabolic cellular functions, but has limited availability in the environment (2, 3). Iron can accept and donate electrons and primarily exists in two states in biological systems, ferric (Fe^3+^) and ferrous (Fe^2+^), which allows iron to bind different ligands. Ferrous iron (Fe^2+^) can generate reactive oxygen species (ROS) through the Fenton and Haber-Weiss reactions (4, 5). At physiological pH, ferrous (Fe^2+^) iron is oxidized to the low solubility ferric (Fe^3+^) state (6). Iron levels are tightly regulated through the production of various binding molecules and transporters such as transferrin, ferritin and ferroportin, which decrease the potential of ferrous iron to generate ROS (4, 7) (**Figure 1A**). This regulation results in an ongoing battle for iron between mammalian hosts and their microbial inhabitants (8–11). Bacteria evolved to improve their odds in this battle by producing an extensive repertoire of siderophores that bind ferric (Fe^3+^) iron (2, 12). These siderophores are recognized for their diverse functionality beyond iron binding, including roles in signaling, virulence, protection against oxidative stress, metal acquisition, and competition with other microbes and their hosts (13–15).

**Figure 2:**
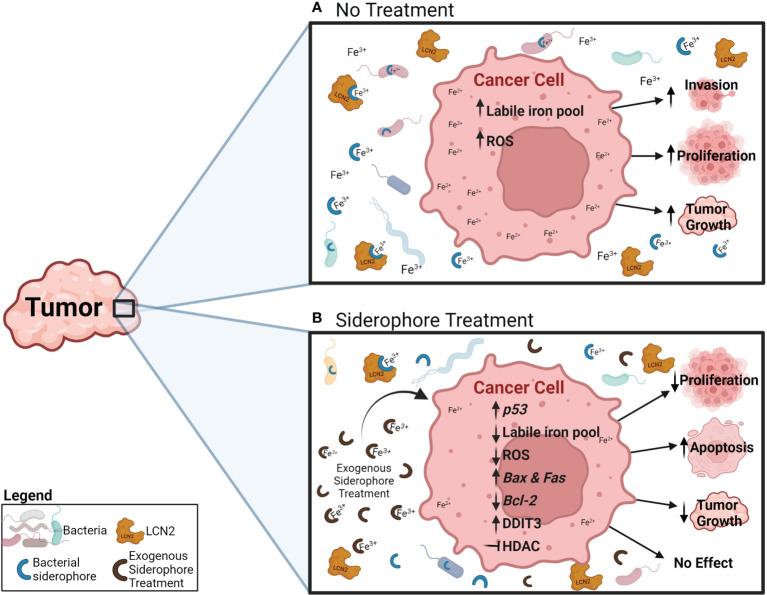
Potential effects and mechanisms of an exogenous siderophore treatment in cancer cells. **(A)** Under normal conditions, cancer cells increase their iron uptake, which increases the iron labile pool (Fe^2+^) and ROS generation, and has been related to increased invasion, proliferation and tumor growth. **(B)** During exogenous siderophore treatment, siderophores bind ferric iron (Fe^3+^) and decrease the levels of free iron available for bacteria, LCN2 and cancer cells. As a result, cancer cells display reduced proliferation and tumor growth, and induction of apoptosis. Proposed mechanisms include: reduction of ferric iron (Fe^3+^) availability, the intracellular iron pool (Fe^2+^), ROS generation and expression of the anti-apoptotic gene *Bcl-2*, increasing expression of *p53* and the pro-apoptotic genes *Bax* and *Fas*, activating the pro-apoptotic pathway through *DDIT3*, and inhibiting HDAC.

Siderophores and iron have become relevant in carcinogenesis because cancer cells demand increased iron concentrations to sustain rapid proliferation, which increases the activity of many iron-binding molecules [transferrin, the transferrin receptor, ferritin and lipocalin 2 (Lcn2)] while decreasing the activity of the cell iron exporter ferroportin (**Figure 1B**) (11, 16–18). Bacterial dysbiosis is also common during cancer (19–22), which could affect siderophore secretion in this disease (**Figure 1B**). Iron accumulation is usually observed in tumors, which has been linked to worse cancer prognosis and increased invasion and metastasis (11, 23) (**Figure 2A**). There is extensive evidence that iron supports various steps of cancer progression, and modulating iron levels has been considered as a promising alternative cancer therapy. Bacterial and synthetic siderophores have been used as iron chelating agents to reduce iron levels in tumors (24) but the function and potential of siderophores in cancer continues to be severely underexplored. Published preclinical data and some clinical studies using siderophores in cancer reported both beneficial and inconsistent results (**Figure 2B**; **Tables 1**, **2**) (35, 46, 68–70), suggesting that their role in cancer warrants further investigation.

**Table 1:** Bacterial siderophores used in cancer research.

Siderophore	Structure 2D	Secreted by	Cancer Type Studied
Deferoxamine (DFO)	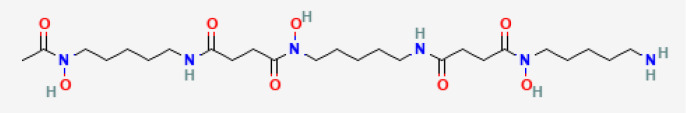 PubChem Identifier: CID 2973 (25)	*Streptomyces* spp	Macrophage (34), leukemia (34, 35), breast cancer (34, 36, 37), hepatocellular carcinoma (34, 38–40), gastric cancer (41), neuroblastoma (42, 43), ovarian (36), epidermoid carcinoma (36).
DFCAF (DFO complex)	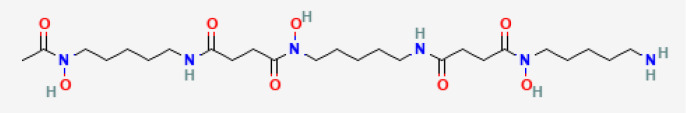 PubChem Identifier: CID 2973 (25) 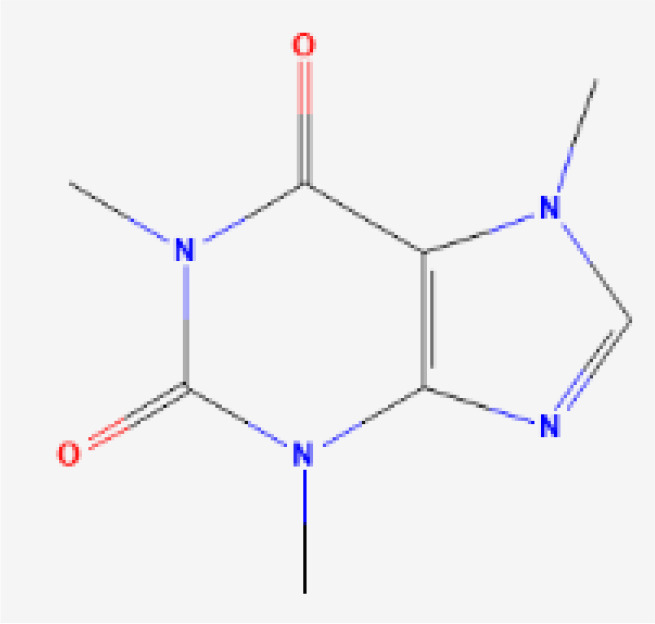 PubChem Identifier: CID 2519 (26)	*Streptomyces* spp	Cancer stem cells (44).
Desferrithiocin (DFT)	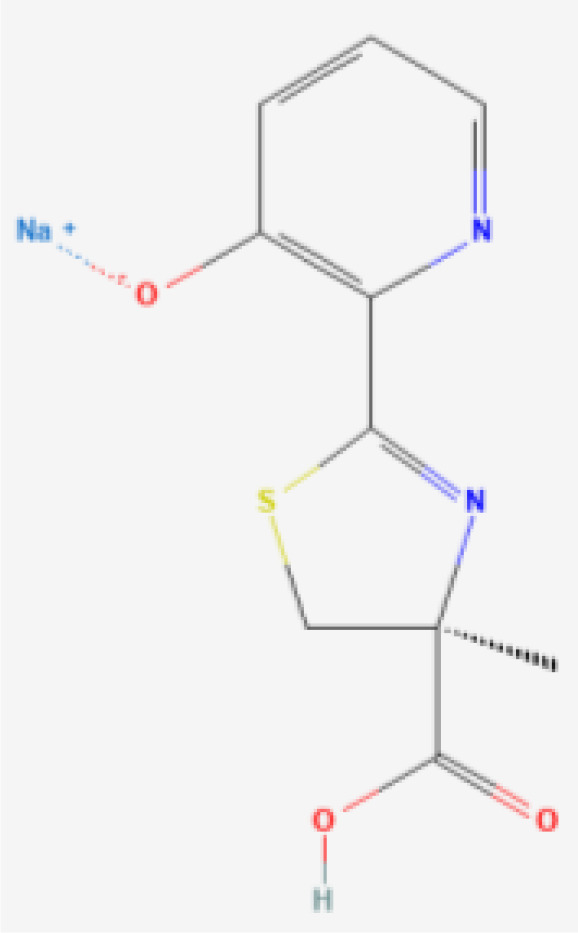 PubChem Identifier: CID 101609363 (27)	*Streptomyces antibioticus* DSM	Hepatocellular carcinoma (45).
Enterobactin	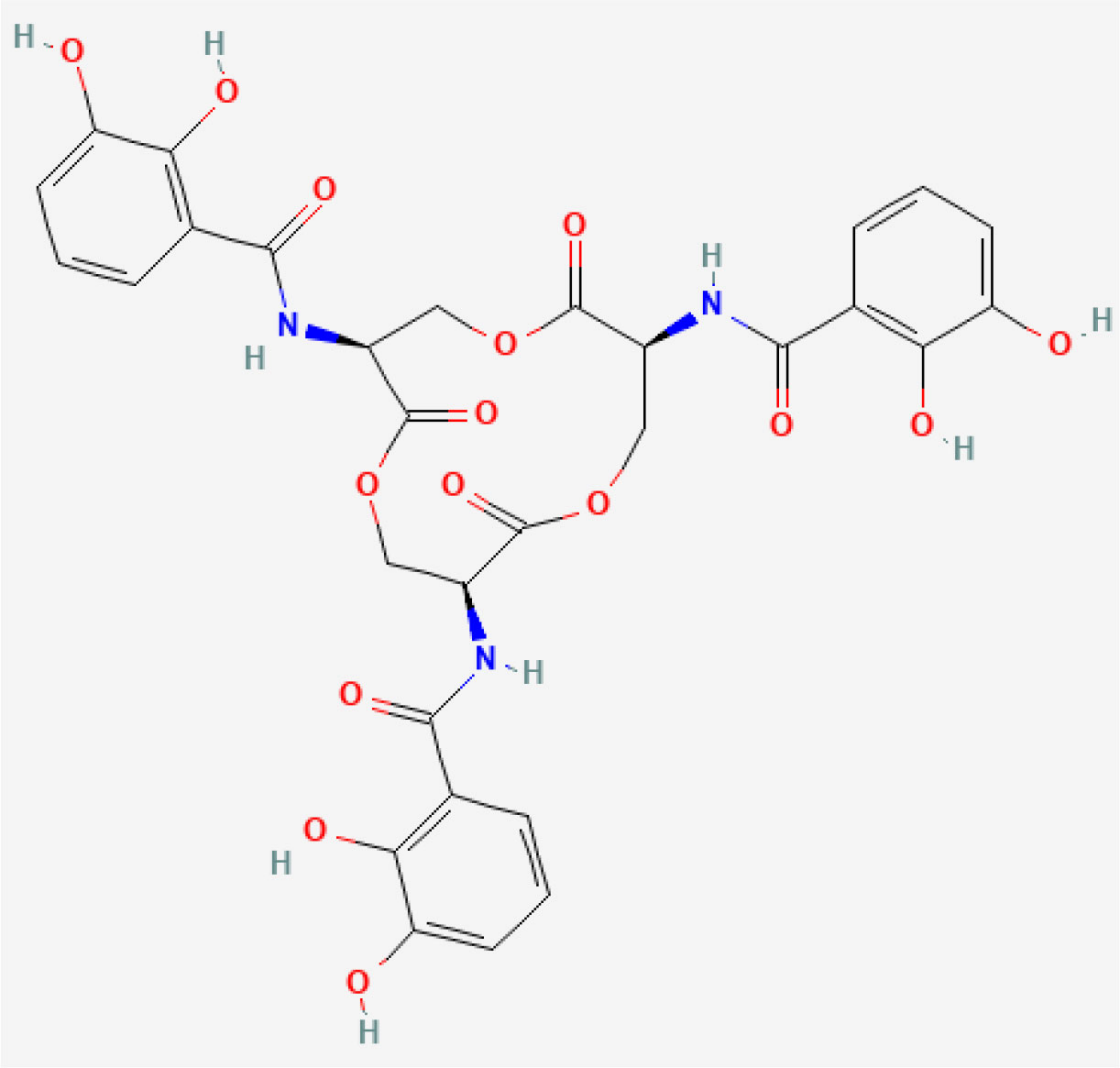 PubChem Identifier: CID 34231 (28)	*E. coli, Salmonella enterica, Shigella dysenteriae and Klebsiella pneumoniae*	Monocyte-derived cancer cells (46).
Ferrichrome	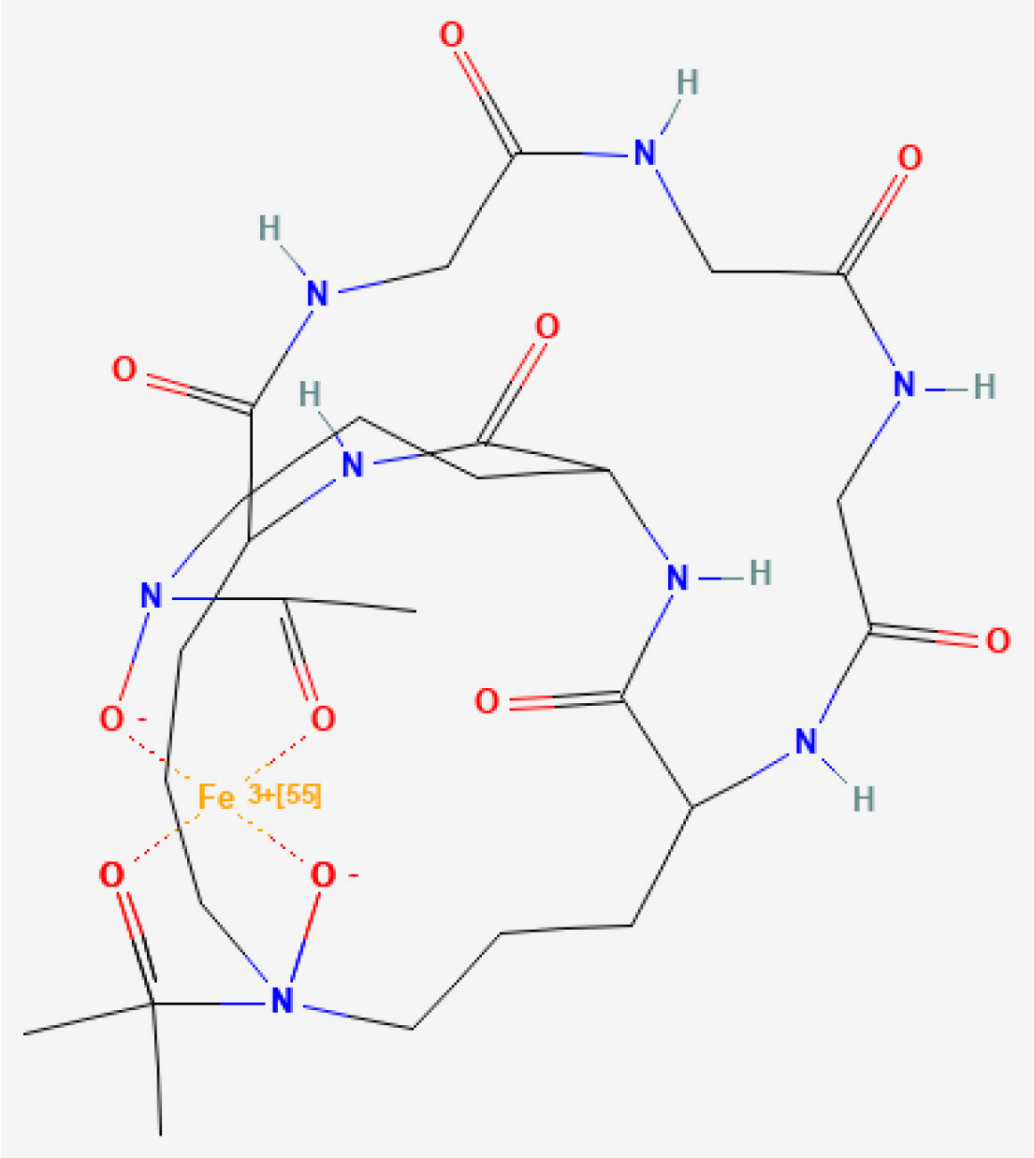 PubChem Identifier: CID 644246 (29)	*Lactobacillus casei*	Gastric (47) and colon cancer (48, 49).
Exochelin-MS	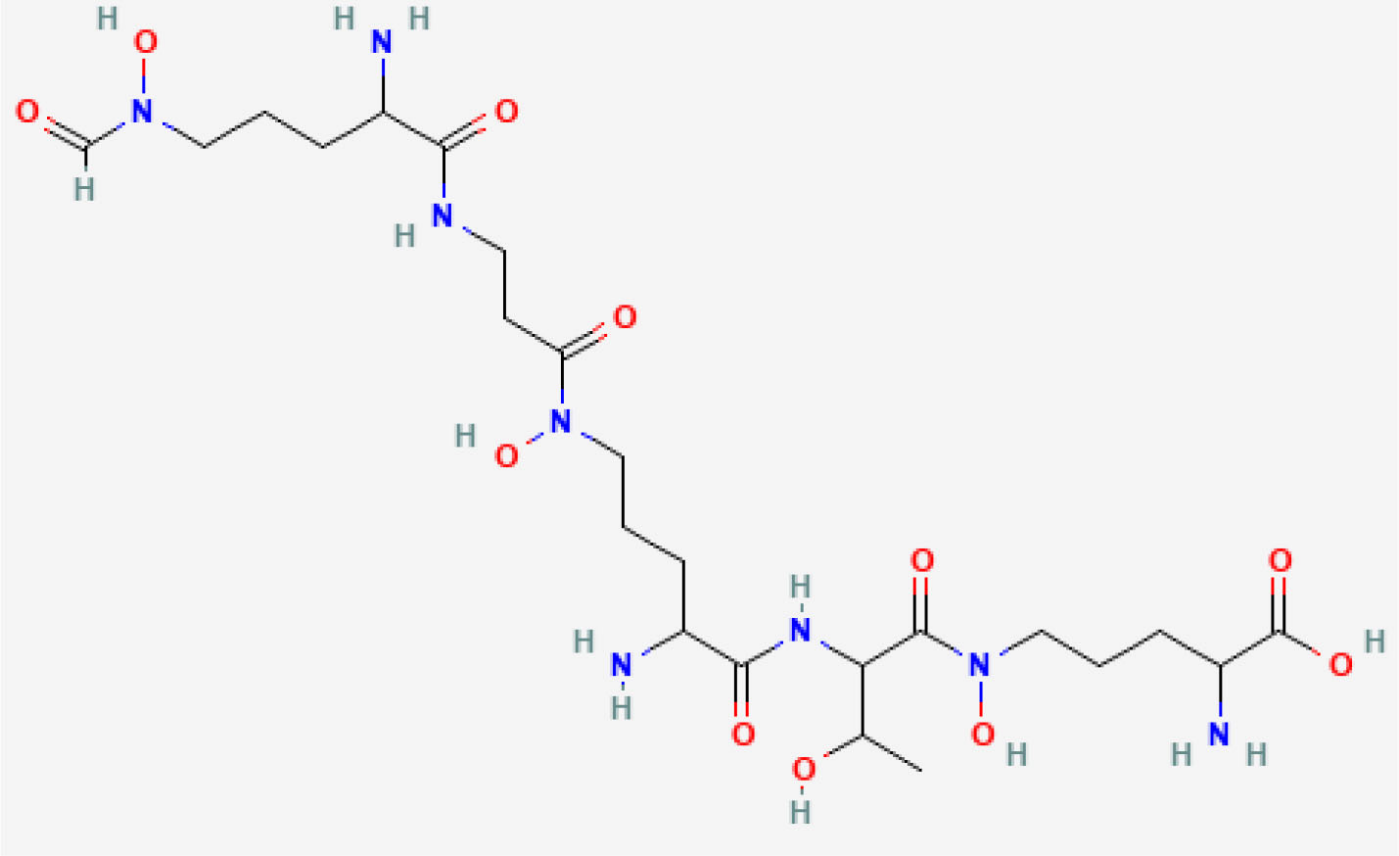 PubChem Identifier: CID 139583168 (30)	*Mycobacterium smegmatis*	Macrophage, liver cancer, leukemia, breast cancer (34).
Mycobactin S	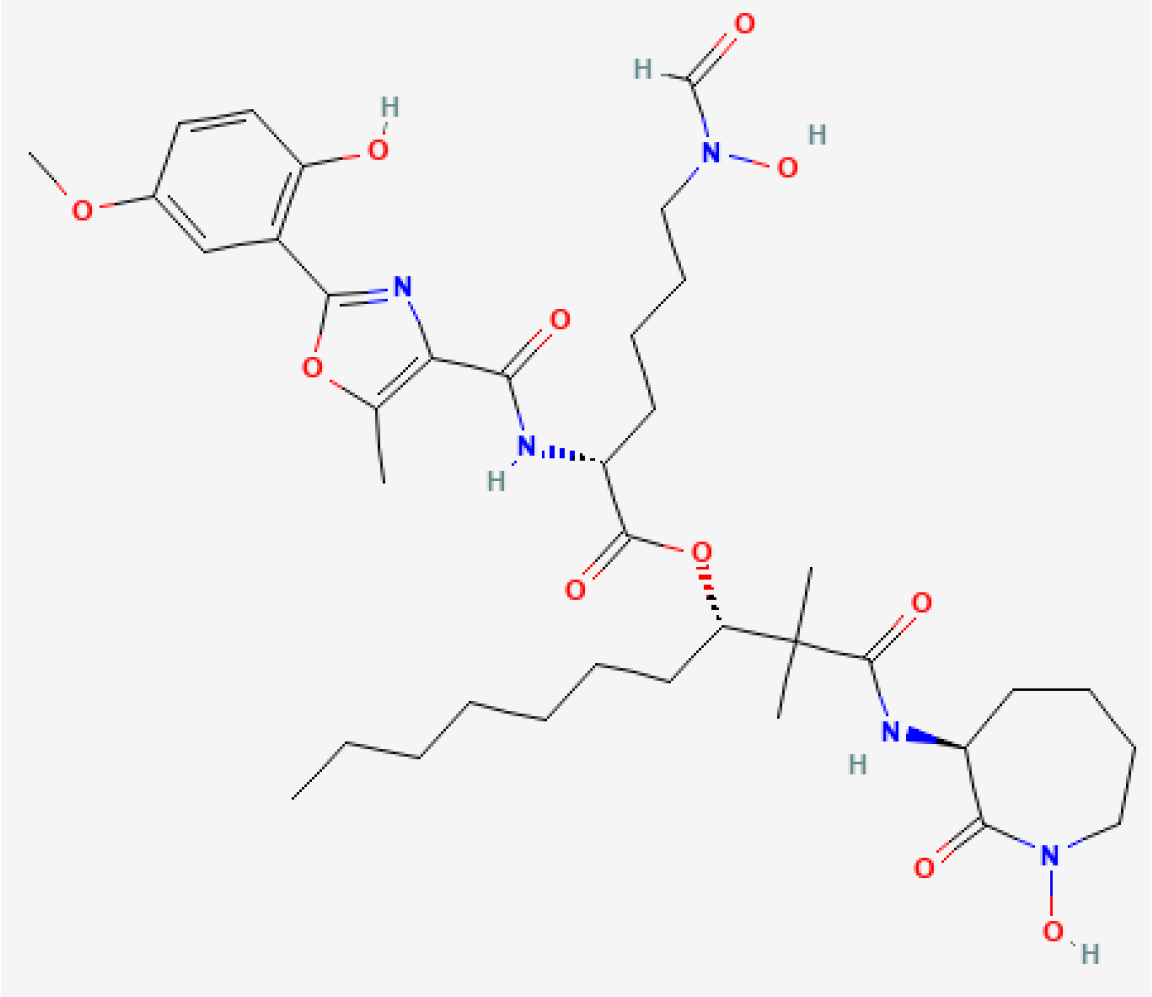 PubChem Identifier: CID 3083702 (31)	*Mycobacterium smegmatis*	Macrophage, liver cancer, leukemia, breast cancer (34).
Amamistatin A	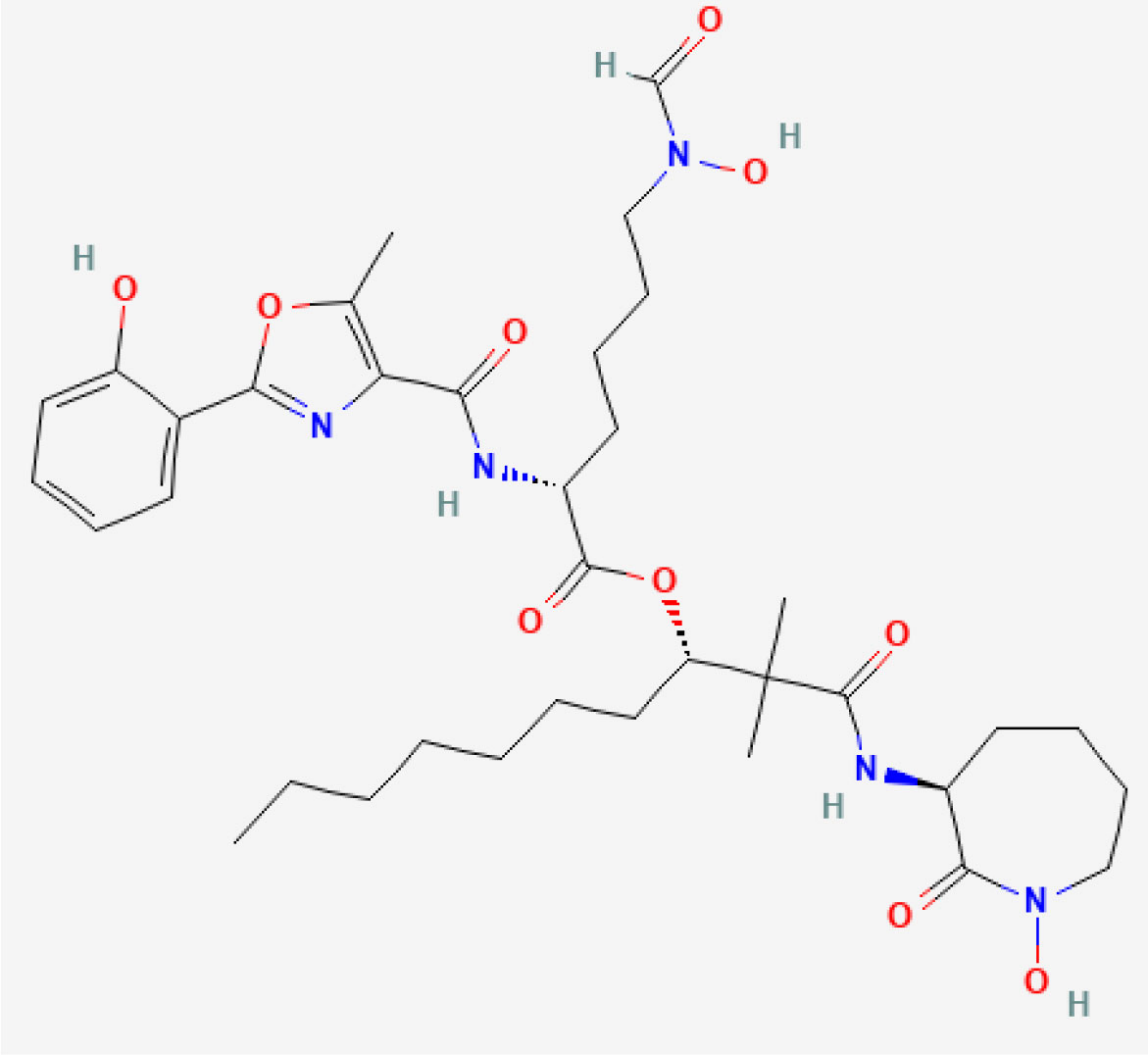 PubChem Identifier: CID 135430484 Amamistatin A (32)	*Nocardia asteroids*	Leukemia, breast, lung, and stomach cancer (50).
Amamistatin B	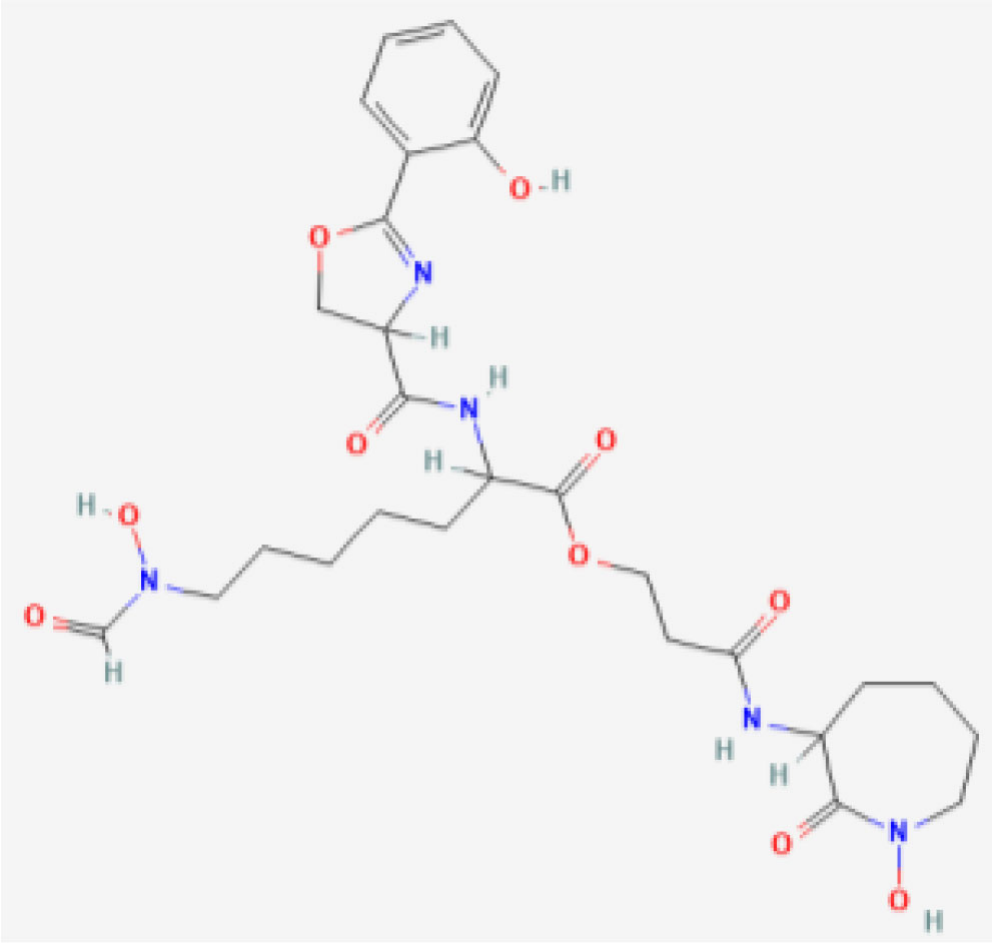 PubChem Identifier: CID 135438025 Amamistatin B (33)	*Nocardia asteroids*	Leukemia, breast, lung, and stomach cancer (50).

**Table 2:** Clinical trials that investigated siderophores and siderophore analogs as cancer therapeutics.

Study title	Age years	Status	Enrollment	Condition	Intervention	Primary Outcome	Intervention Regime	Clinical Trial Number	Source	Refs.
Pilot Study to Assess Hematologic Response in Patients with Acute Myeloid Leukemia or High Risk Myelodysplastic Syndromes Undergoing Monotherapy With Exjade (Deferasirox)	≥18	Completed	25 (estimated)	High risk myelodysplastic syndromes or acute myeloid leukemia	Deferasirox (Exjade, ICL670)	Number of adverse events (time frame: 2 years)	Oral administration	NCT02233504	clinicaltrial.gov	
Deferasirox in Treating Iron Overload Caused by Blood Transfusions in Patients with Hematologic Malignancies	≥18	Completed	16	Leukemia, lymphoma, and 133 more	Deferasirox (Exjade, ICL670)	Changes in mean neutrophil values (measured by lab) for Arm 1 (time frame: baseline up to 6 months)	Once daily, orally, for up to 6 months or until blood counts recover in the absence of disease progression or unacceptable toxicity	NCT01273766	clinicaltrial.gov	
Combination Study of Deferasirox and Erythropoietin in Patients with Low- and Int-1-risk Myelodysplastic Syndrome	≥18	Completed	28	Low and Int 1-risk myelodysplastic syndrome	Deferasirox, erythropoietin alpha	Difference in percentage of patients achieving erythroid response within 12 Weeks, by treatment group (time frame: baseline up to 12 weeks)	Deferasirox dispersible tablet 10 mg/kg/day or deferasirox film-coated tablet (FCT) 7 mg/kg/day in combination with erythropoietin 40,000 units/week (or erythropoietin alone)	NCT01868477	clinicaltrial.gov	
Myelodysplastic Syndromes (MDS) Event Free Survival with Iron Chelation Therapy Study	≥18	Completed	225	Myelodysplastic syndromes	Deferasirox	Event free survival (time frame: Day 1 to end of treatment period, approx. 7 years)	10 mg/kg/day (once daily) for the first 2 weeks of treatment, followed by 20 mg/kg/day (once daily) from Week 2 to end of treatment	NCT00940602	clinicaltrial.gov	(51)
This Study Will Evaluate Efficacy and Safety of Deferasirox in Patients with Myelodysplastic Syndromes (MDS), Thalassemia and Rare Anemia Types Having Transfusion-Induced Iron Overload	≥2	Completed	111	Myelodysplastic syndrome, thalassemia	Deferasirox, ICL670	Changes in ferritin level compared to baseline in patients with transfusion-induced iron overload treated with exjade (time frame: baseline assessment is followed by monthly assessments for up to 1 year)	NA	NCT01250951	clinicaltrial.gov	(52)
Efficacy and Safety of Deferasirox in Patients with Myelodysplastic Syndrome and Transfusion-dependent Iron Overload	≥18	Completed	63	Myelodysplastic syndromes, transfusion-dependent iron overload	ICL670/Deferasirox	To assess iron chelation by comparing serum ferritin values at baseline vs. 52 weeks of treatment with deferasirox (time frame: 52 weeks)	NA	NCT00481143	clinicaltrial.gov	(53)
Evaluating the Efficacy of Deferasirox in Transfusion Dependent Chronic Anaemias (Myelodysplastic Syndrome, Beta-thalassaemia Patients) with Chronic Iron Overload	18–80	Completed	309 (estimated)	Myelodysplastic syndromes, beta-thalassemia	Deferasirox	This study will evaluate the safety and efficacy of deferasirox in transfusion dependent myelodysplastic syndrome, beta-thalassemia major patients with chronic iron overload [time frame: monthly during the therapy and at the end of the treatment (after 9 months therapy)]	NA	NCT00564941	clinicaltrial.gov	
Safety, Tolerability, and Efficacy of Deferasirox in MDS	18–80	Completed	158	Myelodysplastic syndromes, hemosiderosis	Deferasirox	To evaluate the tolerability and safety profile of deferasirox in patients with MDS with post-transfusional hemosiderosis (time frame: baseline assessment and then monthly thereafter)	NA	NCT00469560	clinicaltrial.gov	(54–56)
Study of Deferasirox in Iron Overload from Beta-thalassemia Unable to be Treated with Deferoxamine or Chronic Anemias	≥2	Completed	175	Beta-thalassemia, myelodysplastic syndromes, Fanconi syndrome, Diamond-Blackfan anemia, aplastic anemia	Deferasirox	To evaluate the effects of treatment on the liver iron content	NA	NCT00061763	clinicaltrial.gov	
Magnetic Resonance Imaging (MRI) Assessments of the Heart and Liver Iron Load in Patients with Transfusion Induced Iron Overload	≥18	Completed	118	Hemoglobinopathies, myelodysplastic syndromes, other inherited or acquired anemia, MPD syndrome, Diamond-Blackfan anemia, other rare anemias, transfusional iron overload	Deferasirox	Change in cardiac iron load and cardiac ejection fraction by MRI recorded at baseline and after 53 weeks (time frame: 12 months)	Deferasirox up to 40 mg/kg/day, per os (PO) (orally), dispersible tablets, taken once daily	NCT00673608	clinicaltrial.gov	(57)
Study for the Treatment of Transfusional Iron Overload in Myelodysplastic Patients	≥18	Completed	24	Myelodysplastic syndromes, iron overload	Deferasirox	Number of participants with adverse events and serious adverse events (time frame: up to Week 52)	Deferasirox 20 mg/kg/day OD for 12 months; deferasirox was taken every morning 30 minutes before breakfast	NCT00117507	clinicaltrial.gov	(58)
Evaluation the Effect of Exjade on Oxidative Stress in Low Risk Myelodysplastic Syndrome Patients with Iron Overload	≥18	Completed	21	Myelodysplastic syndrome	Deferasirox (Exjade)	To evaluate the antioxidative effect of Exjade therapy in MDS patients (time frame: one year)	NA	NCT00452660	clinicaltrial.gov	(59)
Effect of Deferiprone on Oxidative-Stress and Iron-Overload in Low Risk Transfusion Dependent MDS Patients	≥18	Completed	19	Myelodysplastic syndrome, iron overload due to repeated red blood cell transfusion	Deferiprone	To evaluate the effect of deferiprone on oxidative stress parameter ROS in iron overloaded and blood dependent patients with MDS (time frame: 4 months)	NA	NCT02477631	clinicaltrial.gov	
A Phase 2 Study of the Efficacy and Safety of Deferasirox Administered at Early Iron Loading in Patients with Transfusion-Dependent Myelodysplastic Syndromes	≥18	Completed	13	Myelodysplastic syndromes	Deferasirox (Exjade)	Primary outcome is time to mean serum ferritin > 1500 μg/l, as measured from the time of initiation using the mean serum ferritin value of 2 consecutive measurements of ferritin, where the first level is >1500 μg/l and CRP is <3 times baseline measurement	Dispersible tablet administered orally, 10 mg/kg/day	2011-004559-38 ISRCTN62162141	EU Clinical Trials Register	
The Efficacy of Deferoxamine in Preventing Nephrotoxicity of Anthracyclins in Pediatric Cancer Patients	2–18	Completed	60	Nephropathy in pediatric cancer patients	Deferoxamine	Blood urea nitrogen, microalbuminuria (urine albumin-to-creatinine ratio), nephropathy sign, serum creatinine, urine creatinine, urine N-acetyl beta glucosaminidase, urine protein	Deferoxamine 10-20,g/kg or 50 mg/kg, once per day *via* IV over 8 hours with or without anthracyclin infusion	IRCT2016021915666N3	World Health Organization	
Deferoxamine for Patients with Advanced Pancreatic Cancer: Pilot Study	≥20	Completed/terminated	10	Pancreatic cancer	Deferoxamine	Safety	NA	JPRN-UMIN000009054	World Health Organization	
The Safety and Efficacy of Deferoxamine for Treating Unresectable Hepatocellular Carcinoma	≥18	Recruiting	100 (estimated)	Unresectable hepatocellular carcinoma	Deferoxamine	Progression free survival in participants with unresectable hepatocellular cancer [time frame: first dose to date of progressive disease or death due to any cause, every 3 cycles up to 36 months (1 cycle=2 weeks)]	Intervention combined with conventional transarterial chemoembolization	NCT03652467	clinicaltrial.gov	
Evaluating Low-Dose Deferasirox (DFX) in Patients with Low-Risk MDS Resistant or Relapsing After ESA Agents	18–100	Recruiting	39	Myelodysplasia	Deferasirox (Exjade)	Percentage of patients without transfusion-dependence at 12 months (time frame: 12 months)	Deferasirox at 3.5 mg/kg/day, orally	NCT03387475	clinicaltrial.gov	
Phase II Study to Evaluate Overall Response in Patients with Higher Risk Myelodysplastic Syndromes (MDS) Treated with Azacitidine with or without Deferasirox	18–80	Terminated	1	High risk myelodysplasia	Azacitidine, azacitidine plus deferasirox	Overall response rate per IWG 2006 criteria (time frame: 1 year)	Azacitidine 75 mg/m^2^, 7 days/28 day cycle SC or IV, deferasirox 10 mg/kg/day	NCT02159040	clinicaltrial.gov	
A Phase II Pilot Study to Assess the Presence of Molecular Factors Predictive for Hematologic Response in Myelodysplastic Syndrome Patients Receiving DeferasiroxTherapy	≥18	Terminated	1	Myelodysplastic syndrome	Bone marrow aspirate, and deferasirox	Fold increase/decrease in gene transcription from baseline bone marrow aspirate of responders versus nonresponders (time frame: 18 months)	Deferasirox; patients are already on commercial deferasirox before entering the study	NCT02663752	clinicaltrial.gov	
Myelodysplastic Syndrome (MDS) Gastrointestinal (GI) Tolerability Study	≥18	Terminated (low enrollment)	12	Myelodysplastic syndrome, transfusional iron overload	Deferasirox (ICL670)	Difference in the frequency of overall newly occurring GI adverse events in the two treatment arms (time frame: 3 months)	Deferasirox 20 mg/kg/day taken in the morning, 30 minutes before food OR deferasirox 20 mg/kg/day taken in the evening, no less than 2 hours after the last food intake or at least 30 minutes before the evening meal	NCT01326845	clinicaltrial.gov	
Azacitidine Plus Deferasirox (ICL670) in Higher Risk Myelodysplastic Syndromes (MDS)	≥18	Terminated (accrual too slow)	1	Myelodysplastic syndromes	Deferasirox (Exjade, ICL670) plus azacitidine	Difference in proportion of patients with hematologic improvement as defined by the IWG criteria^30^ with the addition of deferasirox to azacitidine compared with azacitidine alone in higher risk non-responding MDS patients after 6 cycles of azacitidine (time frame: 6 months)	Azacitidine 75 mg/m^2^ sc daily for 7 days every 28 days for 6 cycles plus deferasirox 10–30 mg/kg/day depending on transfusion needs	NCT02038816	clinicaltrial.gov	
Deferoxamine for Iron Overload before Allogeneic Stem Cell Transplantation	≥18	Terminated (slow patient accrual)	5	Acute myeloid leukemia, acute lymphoblastic leukemia, myelodysplastic syndrome	Deferoxamine	Safety of deferoxamine therapy determined by the number of participants with Grade 3 or higher toxicities (time frame: baseline, 6 months, 1 year)	50 mg/kg/day of deferoxamine as chelation therapy for at least 2 weeks prior to receiving myeloablative transplant, intravenously or subcutaneously	NCT00658411	clinicaltrial.gov	(60)
Deferasirox in Treating Patients with Iron Overload after Undergoing a Donor Stem Cell Transplant	≥18	Terminated(slow accrual of patients)	4	Breast cancer, iron overload, leukemia, lymphoma, multiple myeloma and plasma cell neoplasm, myelodysplastic syndromes, neuroblastoma, ovarian cancer	Deferasirox (Exjade)	Number of patients not completing treatment (time frame: 6 months)	20 mg/kg once daily orally for 6 months	NCT00602446	clinicaltrial.gov	
Treatment of Iron Overload with Deferasirox (Exjade) in Hereditary Hemochromatosis and Myelodysplastic Syndrome	18–80	Terminated (failure to recruit patients with hemochromatosis to the deferasirox arm)	50	Hemochromatosis, myelodysplastic syndromes	Deferasirox (Exjade) and Venesection	Changes from baseline in liver iron concentration and heart iron concentration determined by MRI, and in bone marrow iron content determined by microscopy after treatment with deferasirox (time frame: 0, 6, and 12 months)	Deferasirox tablet (250 mg or 500 mg) dispersed in a drinkable solution, 10 mg/kg/day, once daily for 12 months OR once daily for 2 weeks and thereafter 20 mg/kg/day for 11.5 months, treated with venesection every 8–10 days for 12 months, or until serum-ferritin has been reduced to 50 µg/L	NCT01892644	clinicaltrial.gov	(61–65)
Deferasirox for Treating Patients Who Have Undergone Allogeneic Stem Cell Transplant and Have Iron Overload	≥18	Terminated (low enrollment)	1	Leukemia, lymphoma, and 133 more	Deferasirox (Exjade, ICL670)	Number of patients with elevated labile plasma iron above threshold (0.5 Umol/L) (time frame: at baseline)	Patients receive oral deferasirox once daily for up to 6 months in the absence of unacceptable toxicity, low dose deferasirox on labile plasma iron is also examined	NCT01159067	clinicaltrial.gov	
Deferasirox, Cholecalciferol, and Azacitidine in the Treatment of Newly Diagnosed AML Patients Over 65	65-89	Terminated (low patient accrual)	4	Acute Myeloid Leukemia	Deferasirox (Exjade), cholecalciferol and azacitidine	Complete remission rate (time frame: up to 5 years)	Deferasirox (20 mg/kg/day) on days 1–7 of protocol, repeated every four weeks for 8 cycles given PO; cholecalciferol (4,000 units/day), on days 1–7 of protocol, repeated every four weeks for 8 cycles given PO; azacitidine (75 mg/m^2^ SC or IV administration) on days 1–7 of protocol, repeated every four weeks for 8 cycles	NCT02341495	clinicaltrial.gov	
Deferasirox in Treating Patients with Very Low, Low, or Intermediate-Risk Red Blood Cell Transfusion Dependent Anemia or Myelodysplastic Syndrome	≥18	Terminated (low accrual)	2	Anemia, myelodysplastic syndrome	Deferasirox (Exjade)	Proportion of patients that achieve erythroid hematologic improvement	PO QD; treatment continues for up to 52 weeks in the absence of disease progression or unacceptable toxicity	NCT02943668	clinicaltrial.gov	
The Effect of Deferasirox on Response Rate of Acute Leukemia Patients Not Treated by Standard Chemotherapy Regimens	≥15	Unknown	40	Acute myeloid leukemia, acute lymphoid leukemia	Cytarabine (cytosar) and deferasirox (osveral)	Complete remission (time frame: first month)	Oral deferasirox at 20 mg/kg per day with cytarabine at 20 mg/m^2^, SC, two times a day for 10 days every 30 days for 1 cycle (or cytarabine alone)	NCT02413021	clinicaltrial.gov	(66, 67)
Study of the Outcome of Patients with Acute Myeloblastic Leukemia and Myelodysplastic Syndrome Receiving Iron Chelation Therapy after Allogeneic Hematopoietic Stem Cell Transplantation	≥18	Unknown	150 (estimated)	Myeloid leukemia, myelodysplastic syndromes	Exjade (deferasirox)	Impact of iron chelation on relapse-free survival rate (time frame: at 2 years)	Exjade at 10 mg/kg per day if the ferritin level reached 1000 ng/ml at 6 months after allograft, for a minimum duration of three months and up to 6 months	NCT03659084	clinicaltrial.gov	

Siderophore interactions with the immune system contribute to the ongoing struggle for iron homeostasis. Immune cells enhance the production of siderophore-binding proteins and proinflammatory cytokines (71–73). In response, bacteria upregulate siderophore production and synthesize stealth siderophores to evade host immune defenses which will be described in detail below (3, 72). These siderophore-mediated adjustments in iron availability suggest a link between the immune response and iron accumulation in cancer cells. Insight into the balance between iron, siderophores, and immune function could contribute to a more comprehensive understanding of the role of siderophores in cancer.

This review focuses on the current research investigating the role and therapeutic potential of bacterial siderophores in cancer, highlighting their functions and interactions with the microbiome and the immune system that could be relevant in cancer research. While we provide brief summaries on siderophore functionality and the relationship between iron and cancer, we would like to direct the readers to the following reviews for a deeper understanding of these topics (4, 13, 14, 23). This review also lists the clinical trials that use siderophores and analogs as interventions in cancer. We compare and discuss their settings and regimens to provide a broader perspective of how siderophores are administered to patients, and what factors need to be taken into consideration in cancer. We searched the following databases: clinicaltrials.gov, the European Society of Medical Oncology (ESMO), and the World Health Organization. We used the following search terms: “siderophores” OR “deferoxamine” OR “deferasirox” OR “enterobactin” OR “desferrithiocin” OR “ferrichrome” OR “deferiprone” OR “2,3‑dihydroxybenzoic acid” AND “cancer”. By highlighting the role of these siderophore interactions and how they are used in preclinical cancer models, we aim to promote novel research that leverages these connections in cancer detection and treatment.

## 2 Siderophores As Iron Chelators

Iron is an essential element for nearly all life forms, with critical roles in various biological and metabolic processes for cell survival. Iron availability within aerobic environments is severely limited due to low ferric (Fe^3+^) iron solubility and low ferrous (Fe^2+^) iron availability (2, 3). Essentially all microorganisms, bacteria, fungi, plants, and animals produce siderophores to sequester iron from the environment. Siderophores are high-affinity iron chelators that bind iron to maintain iron levels required for survival (1, 3, 8, 12, 14). Siderophores are small molecules, around 500–1500 Daltons in molecular weight that bind primarily to ferric iron (Fe^3+^) (2) and exhibit high structural diversity (hundreds have been structurally characterized and described) (2, 74). Thus, siderophores are biologically important and have the potential to selectively compete in cellular processes, which makes them an interesting target in cancer research. Understanding their functions and interactions in the microbiome and with the host will be needed to explore their role and potential in cancer.

### 2.1 Mammalian Siderophores

Free ferric iron (Fe^3+^) is tightly regulated in human hosts; most iron is bound to transferrin in serum, or to lactoferrin primarily in secretory fluids, thereby limiting iron availability for bacterial acquisition (8–10). Mammals produce a limited number of siderophores, including 2,5-dihydroxybenzoic acid (2,5-DHBA) (8, 75) and catechols (76, 77), which enable iron acquisition from transferrin, lactoferrin, and the environment **Figure 1A** (71, 77, 78). Synthesis of the mammalian siderophore 2,5-DHBA is catalyzed by the enzyme 3-hydroxybutyrate dehydrogenase-2 (Bdh2) (8). During the innate immune response to lipopolysaccharide exposure, toll-like receptor 4 (TLR4) suppresses Bdh2 through the transcriptional repressor B lymphocyte–induced maturation protein (Blimp-1) (8, 79). This reduces the circulating 2,5-DHBA levels during bacterial infection and limits the host-derived iron complexes available for pathogens to sequester (76, 77, 79). Embedded in the role of iron binding and transport, 2,5-DHBA is involved in intracellular iron homeostasis, erythrocyte maturation, and mitochondrial iron uptake (8, 80, 81). Decreased 2,5-DHBA through reduction of Bdh2 can lead to anemia, cytoplasmic iron accumulation, mitochondrial dysfunction, and potential apoptosis (8, 80, 81). A group of siderophore-binding proteins called lipocalins or siderocalins bind mammalian and bacterial siderophores with specificity for nearly all catecholate siderophores and some carboxylate siderophores (71, 72). Thus, mammalian siderophores, alongside lipocalins and transferrin, have a critical role in mammalian iron homeostasis through intracellular iron balance and mitochondrial function (8, 71, 77, 78, 80). Future work might uncover new siderophores and functionalities within the mammalian iron chelator protein family that could be used to alter iron availability within cancer cells.

### 2.2 Bacterial Siderophores

Bacteria produce siderophores to obtain iron from their hosts or environment and to outcompete other microbes within their environment (3, 14, 82). The ferric uptake regulator (Fur) is a bacterial transcription factor that upregulates siderophore production based on low intracellular iron availability to rapidly acquire iron for metabolic processes and virulence (9, 83, 84). When iron availability is high, Fur suppresses iron acquisition and transport genes, including TonB, thereby preventing iron toxicity and intracellular oxidative stress (83–85). Siderophores enable bacteria to bind ferric iron (Fe^3+^) from their hosts or environment when under low iron availability, and then selectively import iron *via* specific cognate receptors. The specificity of siderophores and siderophore receptors enable microbes to support their own proliferation while competing with other microbial populations for limited iron resources (12, 14). However, there is evidence of both cooperation and competition among bacterial populations for iron, and bacteria can modulate siderophore production in specific hosts or environments (14, 86, 87). Cooperation in bacterial iron uptake involves the production of siderophores that can be taken up by bacterial species other than the initial producer, although there is a correlation between bacterial relatedness and cooperation. Competition in bacterial iron uptake occurs when bacteria that do not produce specific siderophores express receptors that enable siderophore uptake, thereby exploiting the siderophore production of other bacteria. Competition also happens when bacteria secrete specific siderophores for which other bacteria lack the matching receptors for uptake or when the secreted siderophores have higher affinity for iron. Therefore, the bacteria that secreted the specific siderophore (or higher affinity siderophores) decreases the iron available to other species (14, 88). Cooperation and competition has been extensively studied in *Pseudomonas aeruginosa* and its siderophore pyoverdine (PVD) (14, 15, 88–91).

Bacteria can synthesize multiple siderophores with varying iron affinities and minor structural variations, which can enhance iron uptake, reduce the frequency of competitive theft, and improve the competitive advantage over other microbes (3, 12, 82). Several microbes produce stealth siderophores, which are essential in evading the mammalian host innate immune molecule, siderophore binding Lcn2 (3, 72). For example, *E. coli* and *Salmonella* spp. can modify the siderophore enterobactin, which can be neutralized by Lcn2, to form salmochelin, a stealth siderophore that cannot be bound by Lcn2, thereby enhancing their survival when the acute phase response of infection and inflammation has been triggered (2, 3). Enterobactin from *E. coli* is usually associated with a negative impact on iron homeostasis and host health. Mice deficient in Lcn2 have increased susceptibility to *E. coli*-induced septicemia (73). At the same time, enterobactin increases the host iron pool and promotes mitochondrial iron uptake by binding to the ATP synthase α subunit in mammalian cells and *C. elegans* (92 *).* This positive relationship between enterobactin and the host could explain why the host continues to tolerate enterobactin-producing bacteria in their microbiome.

To further showcase the multifunctionality of siderophores, PVD, a siderophore produced by *P. aeruginosa*, can enter *C. elegans* and induce death by acting as a toxin. PVD and *P. aeruginosa* can also disrupt *C. elegans’* mitochondrial homeostasis inducing mitophagy, which is accomplished by the host as a mechanism to resist damages by PVD or *P. aeruginosa* (93). Exogenous iron chelators attenuate *P. aeruginosa*-mediated *C. elegans* killing by limiting bacterial growth. Interestingly, the same iron chelators reduce mitochondrial mass and induce mitochondrial fragmentation in *C. elegans*, and induce mitochondrial turnover and degradation in mammalian cells (93). This same group found that the siderophore PVD (but not pyochelin) was required for *P. aeruginosa* to induce cell death in *C. elegans*, which occurs through hypoxia induction. Similarly, exogenous iron chelators at higher concentrations also trigger a hypoxic response and death in *C. elegans* (94). The flexibility and diversity of bacterial siderophore production indicates that these molecules have important and complex roles in iron acquisition that should be taken into consideration in the context of cancer.

### 2.3 Other Siderophores

Both plants and fungi produce hundreds of unique siderophores. Plant siderophores (phytosiderophores) are most notably secreted by gramineous plants to obtain ferric iron (Fe^3+^), and likely zinc and copper, from soils that are deficient in these metals (95). Fungi utilize siderophores for iron import and can even upregulate bacterial siderophore transporters to compete for iron in the environment (2, 96–98). The focus of this review is bacterial siderophores and their role in cancer; however, the interactions among fungal, bacterial, and host iron acquisition systems may be disrupted in cancer and should be considered.

## 3 Versatile Functions Of Siderophores

Siderophores perform a multitude of functions in addition to iron acquisition. These include roles in signaling, protection against oxidative stress, sequestration of other metals, and siderophore moieties as antibiotics (13). Although iron chelation is the most important and well-characterized role of siderophores, these additional functionalities indicate their biological significance and structural diversity (12). Understanding whether these functions can be applied or studied in the context of cancer will elucidate the potential of siderophores as cancer research targets.

### 3.1 Siderophores as Virulence Factors

Siderophores have been rigorously linked to pathogen virulence (99–104). As mentioned earlier, *Pseudomonas aeruginosa* produces PVD, a siderophore that stimulates its own production along with the virulence factors exotoxin A and lysyl endoprotease through a transmembrane signaling pathway described by Lamont et al. (99, 105). *Klebsiella pneumoniae* strain CG43 carries the virulence plasmid pLVPK, which expresses genes involved in iron acquisition, pathogenicity, capsular polysaccharide synthesis, and genes that confer resistance to lead and tellurite (106). *K. pneumoniae* genes for the siderophores enterobactin, aerobactin, and salmochelin are encoded in the chromosome, the plasmid, or both; aerobactin and salmochelin are associated with hypervirulence (104). Siderophore synthesis promotes iron acquisition in an iron-limited environment, which enhances microbial proliferation and pathogenic virulence during invasion and colonization of the host.

### 3.2 Siderophores as Signaling Molecules

Microbes can use siderophores as intraspecies signaling molecules that are responsive to their environment and modify their own iron acquisition capabilities, and as interspecies signaling molecules between microbial populations across the environment (13). The siderophore PVD from *P. aeruginosa* is produced through a complex signaling pathway, described by Lamont et al. and Beare et al., that also initiates transcriptional activation of the endotoxin A (*toxA*) gene (99, 107, 108). Another signaling mechanism of *P. aeruginosa* involves siderophore-induced upregulation of the TonB-dependent receptors FoxA and FiuA in response to the heterologous siderophores ferrioxamine B and ferrichrome (109). This confers a competitive advantage to *P. aeruginosa* by enhancing iron sequestration from the environment. The *E. coli* TonB-dependent receptor FecA is activated by binding to ferric citrate, and then initiates the transcription of ferric citrate transport proteins to facilitate transport of citrate-bound iron (ferric citrate) into the cytoplasm (110, 111). Some microbial siderophores act as signaling molecules that regulate the production of their own virulence factors, stimulate iron transport, and communicate within and between microbial communities, an important consideration when factoring in disrupted iron homeostasis in hosts with cancer.

### 3.3 Siderophores in Oxidative Stress

ROS are a lethal threat to microbes, and are often synthesized by mammalian polymorphonuclear lymphocytes that utilize iron as part of the innate immune response (112, 113). Siderophores have been implicated in reducing the levels of ROS produced by the host and minimizing oxidative stress through multiple mechanisms (7, 114, 115). For example, the siderophore yersiniabactin, which is produced by some *Yersinia* spp. and other Enterobacteriaceae, inhibits ROS production in human and mouse white blood cells by binding iron more effectively than mammalian lactoferrin and transferrin (7). This subsequently blocks the innate immune system by blocking iron acquisition for the production of ROS, which ultimately reduces oxidative stress in the presence of yersiniabactin (7). *E. coli* produces the enterobactin siderophore, which protects against oxidative stress (116). Enterobactin is internalized and hydrolyzed in the bacteria cytoplasm to relieve oxidative stress by scavenging radicals (116, 117). The role of siderophores in oxidative stress will be discussed in greater detail later.

### 3.4 Metal Sequestration by Siderophores

Siderophores primarily bind iron, but they also can bind other metals based on their major structural group (118). *P. aeruginosa* produces two major siderophores, PVD and pyochelin, which form complexes with Fe^3+^, Ag^+^, Al^3+^, Cd^2+^, Co^2+^, Cr^2+^, Cu^2+^, Eu^3+^, Ga^3+^, Hg^2+^, Mn^2+^, Ni^2+^, Pb^2+^, Sn^2+^, Tb^3+^, Tl^+^, and Zn^2+^, although many of these metals are not efficiently transported into the cell (119, 120). The ability of siderophores to bind and selectively transport specific metals into cells allows these metals to act as cofactors for biological processes (119, 120). However, toxic metals may remain bound to siderophores with limited uptake into cells, while potentially triggering the production of additional siderophores (120–122). Toxic metals that are transported into the microbial cytoplasm can be expelled *via* efflux pumps, thereby enhancing microbial survival in environments containing toxic metals (119, 121–123). This is seen during *E. coli* infections, where *E. coli* secretes the siderophore yersiniabactin as a protective mechanism against copper toxicity (124). Yersiniabactin was found to be a favorable copper (II) ligand that prevents copper (II) reduction to copper (I) and helps *E. coli* resist toxicity during infections (7, 124).

With a different purpose, gallium (Ga^3+^) can replace ferric iron (Fe^3+^) already bound to siderophores (125). Gallium and iron share similar chemical properties, which allows gallium to also bind iron-binding molecules like transferrin and ferritin, and to be taken up by cells through the transferrin receptor (126–129). Gallium and iron differ in their pharmacokinetics and cellular functions, iron is eliminated at a faster rate than gallium and they cannot be interchanged in essential iron-catalyzed reactions (130). The ability of siderophores to bind other metals and interfere with iron metabolism suggests a therapeutic potential for gallium to control and treat bacterial infections (see section titled “*Antibiotic Activity of Siderophores*”). The mechanisms explored here could potentially be applied for siderophores in cancer research. Gallium treatment is already used as a cancer treatment in pre-clinical models, which we describe in the section titled “*Siderophores as Iron Chelating Anticancer Agents*”.

### 3.5 Antibiotic Activity of Siderophores

Targeting siderophore-mediated iron acquisition has become an attractive approach to treat bacteria, as they become more resistant to drug antibiotics. For example, gallium was found to reduce microbial iron uptake and hamper bacterial growth. By binding endogenous siderophores like PVD and pyochelin, gallium can be taken up instead of iron by *P. aeruginosa*, which disrupts iron metabolism and inhibits bacterial growth (131–134). Gallium coupled with the siderophore deferrioxamine is a successful bactericidal therapy against *P. aeruginosa* infection (135). Importantly, the antibacterial activity of gallium on *P. aeruginosa* is greatly influenced by the siderophore ligand type and the bacterial carbon source, which is important to consider when using this mechanism to develop medical therapies (136). Therefore, siderophores may have antibacterial effects by starving pathogens for iron. Similarly, pathogens can control the growth of bacteria by limiting iron acquisition through competition, discussed in the “*Bacterial Siderophores*” section (14). Additionally, siderophores exert antimicrobial activity against competing microbes through the formation of sideromycins (2, 13). Sideromycins are generated by linking a siderophore to an antibiotic component, although naturally occurring sideromycins are limited (2, 13, 137). A number of sideromycins have been synthesized to use as therapeutics against bacterial infections due to their targeted antimicrobial activity (10, 118, 137). *Burkholderia thailandensis* produces the natural sideromycin malleonitrone (138), which exhibits antibiotic activity against Gram-negative bacteria and inhibits *P. aeruginosa* (138). *Streptomyces* spp. produce the sideromycin albomycin, which inhibits protein synthesis and exerts a broad range of antibiotic and antimicrobial activities (2, 13, 137). Nonetheless, pathogenic bacteria can still develop resistance to sideromycins. This is seen in many bacteria mainly through the loss of siderophore receptors (139, 140), and therefore understanding the mechanisms of resistance is vital to develop more effective and targeted treatments. Salmycins, ferromycins, and microcins are sideromycins that exhibit more limited antimicrobial effects, typically against Gram-positive bacteria (13). The antibiotic and antimicrobial activities of sideromycins suggest that the extensive variety of siderophores produced by microbes has biological relevance beyond the acquisition of iron.

### 3.6 Siderophore Interactions With the Immune System

Iron has a key role in immune competency (13). As previously discussed, iron availability within hosts is limited and results in pathogen upregulation of siderophores to confer a survival advantage (83, 141). Iron availability and acquisition are critical for bacterial pathogenesis (103, 142, 143). Increased iron availability in normal and once-immunized mice was associated with lethal infection regardless of *Salmonella typhimurium* virulence, and the observed reduction in mortality of twice-immunized mice was associated with timing of the iron injection in relation to infection (142). Although excess siderophores in normal mice increased mortality, the injection of siderophores into immune mice did not enhance the lethality of infection (142). Siderophores enhance microbial iron acquisition and may induce competition that alters the balance of the microbiome and iron homeostasis in the host (10, 14, 144).

Iron availability within hosts potentiates pathogen infection/proliferation and cancer, so bound iron is essential for immunity. Iron regulation is more tightly controlled during host disease states as a form of nonspecific immunity used to mitigate the proliferation of pathogens or cancer (77, 145, 146). As previously mentioned, Lcn2 represents a method of iron regulation during immune stimulation as part of the acute phase response (72, 77, 147), and can be synthesized by a variety of cells, including neutrophils and epithelial cells (147–149). Mouse Lcn2 was upregulated in macrophages (*in vitro*) and in serum (*in vivo*) *via* TLR4 receptor signaling when stimulated or injected, respectively, with lipopolysaccharide (73). Lcn2 transcription was increased in blood cells, hepatocytes, macrophages, fibroblasts, and endothelial cells of mice infected with *E. coli* H9049, further implicating its role in innate immune function (73). Lcn2 has been extensively reviewed in the literature regarding its relation to cancer (150–155), whereas interactions between siderophores and the immune system have not been as comprehensively studied.

Some siderophores interact directly with the immune system by interfering with host defense mechanisms. For example, the enterobactin siderophore produced by *E. coli* impeded the defensive functionality of neutrophils by inhibiting ROS that prevented the formation of neutrophil extracellular traps (156). Enterobactin also inhibited myeloperoxidase activity by binding directly to the enzyme, thereby preventing an oxidative burst (157). Lcn2 can bind enterobactin and counterbalance its immune-inhibiting activity in ROS production, neutrophil extracellular trap formation, and myeloperoxidase activity (156–158). By binding siderophores, Lcn2 aids in limiting microbial iron acquisition while simultaneously enhancing the innate immune system (157, 158). However, this competition triggers the production of stealth siderophores that cannot be bound by Lcn2 (2, 157), perpetuating the battle for iron and forcing the host to utilize additional defenses. The host defense peptide LL-37 is produced ubiquitously at epithelial surfaces and binds stealth siderophores such as aerobactin and rhizoferrin to defend against pathogenic microbes (159). Siderophores and Lcn2 also have roles in stimulating the immune response (71, 160, 161). Enterobactin enhanced the secretion of Lcn2 and triggered the release of the inflammatory cytokine IL-6 (161). Siderophores and siderophore-Lcn2 complexes also increase IL-8 expression (160, 161). The Lcn2-induced secretion of proinflammatory cytokines IL-6, IL-8, and CCL20 was enhanced through superfluous siderophore iron chelation (161). Immune cells are sensitive to the levels of iron and other metals that modulate signaling, cytokine production, and antimicrobial functionality (162). Siderophores can initiate the innate immune response and modulate the immune response system through various mechanisms related to iron homeostasis. The ability of hosts to respond to siderophores and control iron homeostasis could be crucial for controlling iron availability in cancer.

The potential of siderophores as therapeutics for cancer warrants further investigation, but the complex interactions between siderophores and the immune system could complicate any beneficial effects. Therefore, it is necessary to understand the role of siderophores in iron chelation and immune response mechanisms before developing innovative cancer therapeutics.

## 4 Siderophores And Cancer

### 4.1 Iron and Cancer

There is a complex relationship between iron and cancer, as different cancers display distinct and altered iron regulation and metabolism (11). Health status and associated diseases have an important role in determining the fate of iron in the body and during carcinogenesis. Most solid tumors accumulate iron within the cancer site (11). Tissue-specific transcriptomic analyses showed that excess ferrous iron (Fe^2+^) and H_2_O_2_ undergo Fenton reactions in the cytosol and mitochondria of 14 different cancers (163). This study predicted that cytosolic Fenton reactions increase intracellular pH by producing OH^–^, thereby increasing glycolytic ATP and nucleotide synthesis, which are key mediators of rapid cancer proliferation (23, 164). The Fenton reaction also generates hydroxyl radicals (•OH), which promotes inflammation and metabolic rewiring, dysregulates cell signals, damages lipids in the cell membrane, and can ultimately induce iron-dependent ferroptotic cell death (23).

The relationship between iron and cancer is clear in hereditary hemochromatosis, a genetic disorder that causes increased iron absorption. Hemochromatosis is a known risk factor for hepatocellular carcinoma (HCC) (165, 166), and previous studies investigated the mechanisms underlying hemochromatosis-mediated excess iron accumulation and tumor development (167). Reducing iron levels *in vitro* and *in vivo* suppresses HCC cell growth (168), and iron chelation therapy is advised for patients with hemochromatosis. The evidence suggests that excess iron can induce p53 mutations (169), which are the primary causes for increased risk of HCC along with increased oxidative stress (170).

One of the most widely accepted hypotheses for the increased iron levels in tumors is the need for increased iron to support and sustain the rapid growth and proliferation of cancer cells compared to non-neoplastic cells. Consistent with this proposal, many cancer cells display upregulation of the iron import protein transferrin, its receptor, and the iron storage protein ferritin **Figure 1B** (16, 18, 171, 172). HCC, breast cancer, ovarian cancer, and colorectal cancer (CRC) display increased levels of transferrin receptor and aberrant expression of other iron transport–related proteins such as ferroportin (iron export transmembrane protein) and the divalent metal transporter (DMT1), required for iron uptake into enterocytes and transport across the endosomal membrane once iron is taken up by the cell (173) **Figure 1B** (174–176). Suppression of the transferrin receptor or reduction of intracellular iron levels in breast and ovarian cancers reduces cancer cell proliferation *in vitro*, inhibits tumor growth *in vivo*, and decreases metastases (175, 176). Conversely, increased cellular iron accumulation from iron loading is associated with increased proliferation in CRC cells and decreased mRNA and protein expression of E-cadherin (174). CRC cells can increase motility and invasiveness by reducing E-cadherin expression, suggesting a role for iron in promoting invasion (174). The Carotene and Retinol Efficacy Trial revealed a trend relating higher iron intake with increased risk of clinically aggressive prostate cancer (177). Although there was no significant association between iron intake and overall risk for prostate cancer in this study, it proposes that iron intake could have a prominent role in prostate cancer (177). Iron dysregulation and excess have been implicated in other cancers, including pancreas (178), lung (179), and bladder cancer (180), melanoma (36), and hematological malignancies (181, 182). These studies support the role of iron in increasing cancer risk by promoting cancer cell proliferation, invasion, tumor growth, and inhibiting apoptosis **Figure 2A**.

Dietary iron supplementation can modulate iron availability in the host, thereby increasing iron availability to cancer cells, promoting microbial siderophore-mediated iron acquisition, changing the intestinal microbiome, and potentially increasing inflammation (183–186). In particular, iron fortification increases the growth of enterobacteria over bifidobacteria or lactobacilli, and increases pathogenic *E. coli* in the microbiome of children (187). Most bifidobacteria and lactobacilli do not secrete siderophores and provide a barrier against pathogenic invasion (188, 189), whereas enterobacteria usually secrete siderophores for iron uptake. This demonstrates a powerful role of dietary iron and siderophores in bacterial growth, and the potential for these changes to influence cancer development.

There is extensive evidence, reviewed here (4, 23), that iron supports cancer progression, and that modulating iron levels can be considered a promising cancer therapy. Iron chelators have been tested as an anticancer treatment to reduce iron levels (190). These chelators consist of bidentate, tridentate, and hexadentate ligands that form octahedral complexes with ferric iron (Fe^3+^) (24). Siderophores secreted by bacteria are considered iron chelators, and siderophore-like molecules have been synthesized to mimic the iron chelation activity of bacterial siderophores and tested in preclinical models (**Table 1**) and patients (**Table 2**). Reducing iron levels through iron chelation has successfully reduced proliferation, tumor growth, and metastasis in preclinical models of pancreatic (191), liver (34, 38), gastric (41), breast (37, 192), prostate (193), esophageal (69), and melanoma (36) cancers (**Table 1**; **Figure 2B**); however, the results from the few patient interventions have had conflicting outcomes (**Table 2**). The following sections evaluate cases and studies where siderophores were involved in cancer.

### 4.2 Siderophore Detection in Cancer

There is scarce evidence on whether siderophores can be detected in tumors or in the microbiome of subjects with cancer. A small pilot study in 2017 evaluated the potential of the sputum microbiome for diagnosing lung cancer status and stage (194). The study included 10 patients referred with possible lung cancer; four were diagnosed with lung cancer after one year, and the other six remained cancer free (194). The study investigated differences in the bacterial species present in patients that developed lung cancer and those that did not. Seven bacterial species were found in both cohorts, and from those, five species were more abundant in the patients that developed lung cancer than those that did not, but only the average percentage abundance of *Streptococcus viridans* was significantly higher. The abundances of *Granulicatella adiacens*, *Streptococcus intermedius*, *Mycobacterium tuberculosis*, *Streptococcus viridans*, and *Mycobacterium bovis* were significantly higher in patients that developed lung cancer than those that did not. Of note, *Mycobacterium tuberculosis* and *Mycobacterium bovis* secrete mycobactin siderophores for iron uptake (143, 195). The researchers used metagenomics sequencing and functional alignment analyses to determine that iron siderophore receptors were higher in patients that developed lung cancer than those that did not (194). Since excess iron has been implicated in lung carcinogenesis (179), understanding the reason for sputum microbiome upregulation of iron siderophore receptors in lung cancer is relevant to understand disease progression. This was the only study that identified resident siderophores or their receptors in cancer. Future studies should characterize these molecules to further understand tumor-microbiome interactions.

### 4.3 Siderophores in Cancer Treatment

Natural and synthetic siderophores have been extensively studied as potential therapies and therapy vehicles for different cancers due to their iron chelating capabilities (**Table 1**; **Figure 2B**). These approaches are discussed in the following sections.

#### 4.3.1 Siderophores as Iron Chelating Anticancer Agents

One of the most popular bacterial siderophores in cancer therapy research is deferoxamine (Desferal, desferrioxamine, desferrioxamine-B, or DFO), which is a water-soluble trihydroxamate hexadentate siderophore secreted by many *Streptomyces* species (196, 197). DFO is commercially available and has been used to treat iron overload diseases (70, 198–200). In leukemia, DFO promotes apoptosis *in vitro* by upregulating the tumor suppressor gene *p53* and the pro-apoptotic genes *Bax* and *Fas*, and reducing the expression of the anti-apoptotic gene *Bcl-2* (35). DFO alone and in combination with doxorubicin chemotherapy inhibited breast tumor growth in xenograft mouse models (37). When combined with doxorubicin, DFO inhibited the cardiotoxic side effects that commonly occur in doxorubicin therapy without compromising the treatment efficacy, suggesting that siderophores have a beneficial role as an adjunct treatment with chemotherapy (37). DFO administration during the initial stages of tumor formation in a subcutaneous xenograft mouse model of HCC regressed or slowed tumor growth due to a decrease in intracellular iron concentration (**Figure 2B**; **Table 1**) (39).

Most preclinical studies demonstrated the antitumor effects of DFO. By contrast, studies in cancer patients had conflicting outcomes, with many clinical trials terminating due to difficulties in patient enrollment (**Table 2**). An early clinical study of ten children with recurrent neuroblastoma (NB) after 1–3 treatment regimens administered continuous IV DFO infusion at 150 mg/kg/day for five consecutive days every other week (42). Within one month from the initiation of therapy, nine patients had progressive disease, and one patient had stable disease. This study concluded that the selected dosage and interval of DFO was ineffective as a single therapy for NB patients. Higher DFO doses such as 240 mg/kg/day have serious adverse effects and are not recommended for use (42). Another clinical trial of ten children with unresectable NB administered DFO at 150 mg/kg/day followed by preoperative chemotherapy, surgery and postoperative chemotherapy (43). There were three complete responses, six partial responses, and one minor response. Nine out of ten patients underwent complete remission following surgery, and the tenth patient underwent complete remission after postoperative chemotherapy. The authors concluded that the DFO regimen used was effective in achieving complete tumor resection (43). A more recent study of ten adult patients with advanced HCC, who did not have a response to hepatic arterial infusion chemotherapy, were administered 10–80 mg/kg of DFO during 24 hours on alternate days an average of 27 times (40). The overall response rate and the 1-year cumulative survival for these patients was 20% (40), indicating that DFO treatment was not successful in the majority of cases. Only four of the clinical trials registered, that used siderophores and iron chelation therapy in cancer, used DFO for the intervention (**Table 2**). One of these studies focused on patients with leukemia and myelodysplastic syndrome and reported low accrual (*n*=5) due to the need for home administration of deferoxamine (50 mg/kg/day) and the considerable intervention window (60). All patients enrolled had iron overload and were scheduled to undergo myeloablative allogeneic hematopoietic stem cell transplantation (60). No serious adverse side effects were reported and serum ferritin decreased after the treatments, but liver iron content remained unchanged. The estimated progression-free survival after transplantation was 100% (60), but the authors are not able to draw any reliable conclusions due to the low number of patients enrolled. The other three clinical trials are in pediatric cancers with nephropathy, HCC patients, or pancreatic cancer patients. The HCC trial is currently recruiting while the pancreatic cancer trial was completed/terminated and did not provide the DFO dosage or preliminary results. Conclusions from the clinical studies described here are limited and should be taken with caution due to the low enrollment, lack of standardized dose, and absence of appropriate control groups.

DFO is a hydrophilic molecule that presents many disadvantages including poor membrane permeability, poor oral viability, and a short plasma half-life of ~12 minutes (201, 202). This requires that DFO is continuously administered subcutaneously or intravenously (68, 203) according to a rigorous infusion regime 8–12 hours/day 3–7 days/week (204, 205). As expected, this administration method has a compliance of <50% (206). The inconsistent success of DFO as an antitumor agent has encouraged the design of more effective iron chelators, with particular focus on chelator lipophilicity, membrane permeability, and selective antitumor activity.

To improve the efficacy of DFO as a cancer therapy, researchers analyzed the effects of a synthesized DFO variation bound to caffeine called desferrioxamine-caffeine dimer (DFCAF), which has greater cell permeability than DFO alone (44). DFCAF cytotoxicity was tested against cancer stem cells (CSCs, the source of biological variability within a tumor), which often determine neoplasm resistance to traditional therapies. CSCs give rise to diverse tumor cell populations and can initiate metastasis, and therefore demand a high intake of nutrients such as iron (44). DFCAF conferred greater ability to sequester iron within breast CSCs, which was measured as the concentration of an intracellular iron storage protein remaining after treatment. The CSCs also had reduced viability, reversed epithelial-to-mesenchymal transition, and increased clearance after DFCAF treatment *in vitro*.

Another bacterial siderophore that has been studied is desferrithiocin (DFT) (**Table 1**). DFT is a tridentate natural siderophore from *Streptomyces antibioticus* DSM (207), which displays antineoplastic activity in HCCs *in vitro* and has high oral effectiveness (45). However, it also confers severe nephrotoxicity, which makes it unsuitable for chelation therapy.

Enterobactin is produced by Gram-negative Enterobacteriaceae (208) (*E. coli*, *Salmonella enterica*, *Shigella dysenteriae*, and *Klebsiella pneumoniae*) and is reported to have anticancer properties (**Table 1**). This siderophore does not have polar properties and is permeable across the cell membrane. Although there is a lack of evidence relating this siderophore to cancer, enterobactin displays anticancer properties by disrupting the generation of ROS and disturbing the homeostasis of the labile iron (redox-active iron that can be chelated) pool *in vitro*. (**Figure 2B**) (46) Enterobactin showed efficacy in selectively chelating iron in monocyte-derived cancer cell lines and refraining from sequestering iron pools in bone marrow–derived macrophages (46). Iron-bound enterobactin exhibits cytotoxic capabilities in rapidly dividing cell lines, whereas unbound enterobactin does not display cytotoxic effects. The observed increase in intracellular Lcn2 concentrations in noncancerous cells could reflect the reduced tendency of enterobactin-induced cytotoxicity in these cells, whereas the lower Lcn2 levels in cancerous cells may allow the increased free enterobactin to exhibit its anticancer properties (46). Lcn2 levels are usually higher in many types of cancers (152) (not the case in this study), which might prevent these results from being translated to other tumor types.

Ferrichrome is another bacterial siderophore with anticancer properties (**Table 1**). Ferrichrome originates from the common probiotic strain *Lactobacillus casei* (47), and was studied for potential cytotoxic effects on gastric and colon cancer cell lines and xenograft mouse models (47, 48). Unbound ferrichrome has antitumor activity through activation of the pro-apoptotic pathway c-Jun N-terminal (JNK)-DNA damage–inducible transcript 3 (DDIT3) (**Figure 2B**). In this study, iron-bound ferrichrome did not display the same antitumor suppression activity. The authors suggested that the mechanism of antitumor activity in ferrichrome is likely located in the iron-chelation site (47). Another study used a xenograft model of CRC and reported similar results for the tumor suppression activity of unbound ferrichrome through upregulated DDIT3 (49). Ferrichrome treatment did not alter serum iron levels in mice, suggesting that it does not necessarily reduce systemic iron levels (49).

A recent study investigated the effects of three bacterial siderophores on the proliferation of malignant and non-malignant mouse and human cell lines: exochelin-MS (Exo-MS), mycobactin S (MBS), and deferoxamine B (DFO) (34). Exo-MS and MBS are water-soluble and lipid-soluble siderophores, respectively, and both are produced by *Mycobacterium smegmatis*. DFO is a water-soluble siderophore. Exo-MS inhibited the growth of mouse cancer cell lines, but not human cancer cell lines (34). DFO inhibited the growth of mouse cancer cells and human breast and leukemia cancer cells (34). MBS decreased the survival of human liver cancer cells, which was not observed with DFO or Exo-MS. The authors suggested that conjugating water-soluble siderophores with lipid molecules could increase their effectiveness against tumor cells (34).

The antitumor effects of siderophore-like molecules derived from bacteria have also been investigated. Amamistatin A and B were isolated from the actinomycete *Nocardia asteroids*, and have similar structures as mycobacterial siderophores. Amamistatin A displayed antiproliferative effects against the human tumor cell lines MCF-7 breast, A549 lung, and MKN45 stomach. Amamistatin A and B displayed cytotoxicity against mouse lymphocytic leukemia cells (50). The authors suggested that the antitumor activity of amamistatins could be explained by their iron chelating properties, similar to the bacterial siderophores. Amamistatins might act as histone deacetylase (HDAC) inhibitors through their N-formyl hydroxylamine or retrohydroxamate moiety. HDAC inhibition prevents tumor growth (209), and the retrohydroxamate ligand has been utilized in small molecule HDAC inhibitors (210), suggesting that amamistatins may be useful in cancer therapy and warrant further investigation.

Since siderophores are also able to bind gallium, which prevents the growth of pathogenic bacteria, research has expanded to understand this interaction in cancer. Gallium can be taken up by tumors exhibiting antineoplastic activity (211, 212). In human lymphoma cells, gallium induced cell death by activating pro-apoptotic Bax and inducing mitochondria-generated ROS (168). Gallium nitrate is approved by the FDA and is being studied in clinical trials to treat cancers like Non-Hodgkin’s Lymphoma, where it had favorable results (213). Cancer patients treated with gallium have a disrupted iron metabolism (214) which indicates that the antitumor function of gallium might be due to iron-related effects. However, the mechanism of action in cancer is still not clear, and patients treated with gallium are at higher risk of iron deficiency and complications like anemia. Continuous treatment with gallium nitrate promotes the development of resistance, which is accompanied by changes in iron trafficking and metabolism (134). Interestingly, other types of gallium, like gallium maltolate, can still inhibit the growth of lymphoma cells that become resistant to gallium nitrate (215). Since some bacteria can become resistant to sideromycins, it will be important to assess whether bacterial and synthetic siderophores could generate resistance in cancer cells.

Recent research focus has shifted to the development of synthetic iron chelators with superior pharmacological properties and similar preclinical results (211, 212), that avoid the adverse effects of siderophores in humans. Optimal iron chelators for cancer treatment must be readily absorbable, with a long-half life in the blood and higher affinity for iron over other metals, although we have reported the benefits of siderophores being able to bind other metals. Selectivity for or against cancer cells is also an important characteristic that should be investigated to avoid systemic iron deprivation when it is not needed (212, 216). The synthetic iron chelator thiosemicarbazone-24 (TSC24) suppressed human HCC tumor growth by disrupting iron homeostasis, reducing available iron, and triggering cell cycle arrest and apoptosis without any apparent host toxicity (168). Other synthetic iron chelators currently in preclinical studies as anticancer agents include deferiprone (193) and deferasirox (191, 192, 217), which are also in clinical trials to treat cancers related to iron overload (**Table 2**).

All clinical trials using iron chelation as a cancer therapy are summarized in **Table 2**. A number of bacterial siderophores and siderophore analogs are studied in preclinical studies; however, only DFO, deferasirox (synthetic), and deferiprone (synthetic) are undergoing clinical trials. Deferasirox is more commonly used than DFO, probably due to its more convenient oral administration. None of these clinical trials are investigating the effects of chelators/siderophores in solid tumors (other than one HCC and one pancreatic cancer study without results), whereas most of the research in preclinical studies is focused on tumors. All conditions and diseases studied in the clinical trials involve systemic iron overload, including beta-thalassemia, leukemia, and myelodysplastic syndrome (which requires increased blood transfusions). The clinical studies are not reflective of the preclinical data generated in mouse models and *in vitro* cell cultures, where siderophores are studied for tumor treatment regardless of systemic iron overload. A potential reason for this is that these chelators act systemically and do not specifically target the tumor. Therefore, other tissues could be damaged and anemia could be developed by a drastic reduction in iron levels. Future studies should address chelator targeting for non-systemic reduction in tumor iron levels and obtain further evidence to increase the translation of these studies to the clinic.

#### 4.3.2 Siderophores as Mediators of Drug Delivery

Siderophores and their analogs are also used for iron transport–mediated drug delivery through sideromycins (Trojan horse antibiotics). Microbes can develop antibiotic resistance by altering permeability barriers, altering the drug target binding sites, synthesizing enzymes that destroy antibiotics, and developing mechanisms to transport antibiotics out of the cell before they induce damage (218). Siderophores can evade membrane-associated drug resistance because microbes require nutrient uptake from the environment for survival, which enables a route and target of drug delivery. Siderophores were investigated as a drug transport agent to overcome antibiotic resistance and target pathogenic bacteria. Successful results in targeting antibiotic-resistant bacteria suggested that this mechanism could be used to target cancer cells. This seems to be a promising proposition, as siderophore-iron complexes can bind Lcn2 and travel in circulation (219), and iron-loaded Lcn2 can be taken up by cancer cells where Lcn2 is commonly upregulated (220), creating a pathway for the implementation of a modified “Trojan horse” drug delivery.

A recent study conjugated the siderophore PVD from *P. aeruginosa*, a mixed-type siderophore with hydroxamate and catecholate groups, to synthesize superparamagnetic iron oxide nanoparticles (SPION) (221). The PVD-iron complex has strong binding and high stability, which protects it from hydrolysis and enzymatic degradation. PVD-SPION was covalently conjugated to a mucine 1 aptamer (MUC1_Apt_) and loaded with doxorubicin, which is used in chemotherapy. MUC1_Apt_ is one of the most studied aptamers as it specifically recognizes the mucin 1 (MUC1) protein that is strongly upregulated in most cancer cell surfaces, which makes it a great target in cancer (222). The investigators showed that the PVD-SPION-MUC1_Apt_ complex was successfully taken up by cancer cells, and conferred tumor inhibitory growth effects and improved survival in mice bearing C26 colon carcinoma (221). This complex also served as a diagnostic agent that improved contrast at the tumor site in magnetic resonance images (221). This study is one of the first to show the potential of siderophores as chemotherapy delivery agents and diagnostic tools in cancer.

#### 4.3.3 Risks of Siderophore Treatment

While siderophore treatment in cancer has shown some beneficial results in pre-clinical models, and some siderophores and analogs have been used in clinical trials, additional studies are needed to determine side effects or potential risks of these interventions. Understanding siderophore functions in mammalian cells will help assess the risk of siderophore implementation in cancer. We previously mentioned that iron chelation reduces mitochondrial mass and induces mitochondrial fragmentation in *C. elegans*, and that it promotes mitochondrial degradation in mammalian cells (93) In addition, iron chelation induces *C. elegans’* death through hypoxia (94). Iron chelation therapy is commonly used to lower systemic iron levels in iron overload diseases (202), and data from the literature suggest that chelation therapy could also enhance iron absorption, which might be undesirable in the context of cancer (223). Therefore, investigating the effects of siderophore therapy on the microbiome, the mitochondria (in not only cancer cells, but also other cells of the body) and systemic and cellular iron levels, will be vital when evaluating the feasibility of these interventions.

Another factor to consider is whether cancer cells can become resistant to siderophore treatment, as bacteria are known to become resistant to sideromycins by losing their siderophore receptors (139, 140), and to gallium nitrate treatment (134). Moreover, it is supported that cancer cells employ Lcn2 to collect extracellular iron to support cancer growth in renal cell carcinoma (220) and leptomeningeal metastasis (224). Thus, understanding the function of Lcn2 as a result of a siderophore treatment will further elucidate additional risks related to these treatments.

## 5 Conclusions

Siderophores are secreted by many organisms including bacteria, to sequester essential iron from the environment to sustain their growth. This function has gained interest in cancer research because excess iron availability is linked to increased cancer risk, and cancer cells require higher iron levels to sustain their rapid proliferation and growth **Figure 1** (71, 225). Therefore, bacterial siderophores and siderophore analogs are being utilized to chelate iron and prevent it from being taken up by cancer cells, which inhibits the proliferation and growth of many different cancers in preclinical studies **Figure 2**.

Siderophores also function as signaling molecules that can lead to the production of virulence factors and the regulation of siderophore synthesis. Siderophores can interact with the immune system, mitigate oxidative stress, protect microbes from ROS and bind other metals in addition to ferric iron (Fe^3+^). Finally, siderophores can form sideromycins and have antimicrobial effects against other microbes in the environment. Each of these described siderophore functions does not fully consider the interactions between microbes in a competitive environment, and how these functions may be enhanced or suppressed through these interactions.

There is still much to learn about the effects and interactions of siderophores in cancer. Most of the preclinical and clinical studies use inconsistent dosages, conditions, and safety and outcome measures, which reduce their translational value for additional studies in patients. Data regarding endogenous siderophores in cancer and related changes to the microbiome are also lacking. Additional studies are needed in these areas to assess the potential efficacy of siderophores in cancer detection and therapeutics. This review discussed bacterial siderophores and analogs that have potential benefits for cancer therapy, but even less is known about mammalian or fungal siderophores in cancer and immunity. It will be crucial to map the relationships between siderophores, iron, immune function, and cancer to develop stable, effective, and targeted therapeutics. These efforts should leverage the functions of siderophores and translate the outcomes to the clinical setting.

## Author Contributions

VP-G: study concept and design, development of methodology, drafting of initial manuscript, writing, review, and/or revision of the manuscript, administrative, technical, or material support, and final approval of the version to be submitted. KC: study concept and design, development of methodology, drafting of initial manuscript, writing, review, and/or revision of the manuscript, administrative, technical, or material support, and final approval of the version to be submitted. TS: drafting of initial manuscript and writing, review, and/or revision of the manuscript. ZC-M: study concept and design, development of methodology, drafting of initial manuscript, writing, review, and/or revision of the manuscript, administrative, technical, or material support, final approval of the version to be submitted, and study supervision.

## Funding

This publication was supported by The National Cancer Institute (NCI) R01CA223204 (ZC-M) and the Pelotonia Fellowship Program (VP-G and TS). The content is solely the responsibility of the authors and does not necessarily represent the official views of the National Institutes of Health. Any opinions, findings, and conclusions expressed in this material are those of the author(s) and do not necessarily reflect those of the Pelotonia Fellowship Program or The Ohio State University.

## Conflict of Interest

The authors declare that the research was conducted in the absence of any commercial or financial relationships that could be construed as a potential conflict of interest.

## Publisher’s Note

All claims expressed in this article are solely those of the authors and do not necessarily represent those of their affiliated organizations, or those of the publisher, the editors and the reviewers. Any product that may be evaluated in this article, or claim that may be made by its manufacturer, is not guaranteed or endorsed by the publisher.
